# Artificial intelligence-based decision support software to improve the efficacy of acute stroke pathway in the NHS: an observational study

**DOI:** 10.3389/fneur.2023.1329643

**Published:** 2024-01-18

**Authors:** Kiruba Nagaratnam, Ain Neuhaus, James H. Briggs, Gary A. Ford, Zoe V. J. Woodhead, Dibyaa Maharjan, George Harston

**Affiliations:** ^1^Stroke Medicine, Royal Berkshire NHS Foundation Trust, Reading, United Kingdom; ^2^Stroke Medicine, Oxford University Hospitals NHS Trust, Oxford, United Kingdom; ^3^Brainomix Limited, Oxford, United Kingdom; ^4^Radcliffe Department of Medicine, University of Oxford, Oxford, United Kingdom

**Keywords:** stroke, artificial intelligence, endovascular thrombectomy, clinical outcome analysis, door-in-door-out time

## Abstract

**Introduction:**

In a drip-and-ship model for endovascular thrombectomy (EVT), early identification of large vessel occlusion (LVO) and timely referral to a comprehensive center (CSC) are crucial when patients are admitted to an acute stroke center (ASC). Several artificial intelligence (AI) decision-aid tools are increasingly being used to facilitate the rapid identification of LVO. This retrospective cohort study aimed to evaluate the impact of deploying e-Stroke AI decision support software in the hyperacute stroke pathway on process metrics and patient outcomes at an ASC in the United Kingdom.

**Methods:**

Except for the deployment of e-Stroke on 01 March 2020, there were no significant changes made to the stroke pathway at the ASC. The data were obtained from a prospective stroke registry between 01 January 2019 and 31 March 2021. The outcomes were compared between the 14 months before and 12 months after the deployment of AI (pre-e-Stroke cohort vs. post-e-Stroke cohort) on 01 March 2020. Time window analyses were performed using Welch’s t-test. Cochran–Mantel–Haenszel test was used to compare changes in disability at 3 months assessed by modified Rankin Score (mRS) ordinal shift analysis, and Fisher’s exact test was used for dichotomised mRS analysis.

**Results:**

In the pre-e-Stroke cohort, 19 of 22 patients referred received EVT. In the post-e-Stroke cohort, 21 of the 25 patients referred were treated. The mean door-in-door-out (DIDO) and door-to-referral times in pre-e-Stroke vs. post-e-Stroke cohorts were 141 vs. 79 min (difference 62 min, 95% CI 96.9–26.8 min, *p* < 0.001) and 71 vs. 44 min (difference 27 min, 95% CI 47.4–5.4 min, *p* = 0.01), respectively. The adjusted odds ratio (age and NIHSS) for mRS ordinal shift analysis at 3 months was 3.14 (95% CI 0.99–10.51, *p* = 0.06) and the dichotomized mRS 0–2 at 3 months was 16% vs. 48% (*p* = 0.04) in the pre- vs. post-e-Stroke cohorts, respectively.

**Conclusion:**

In this single-center study in the United Kingdom, the DIDO time significantly decreased since the introduction of e-Stroke decision support software into an ASC hyperacute stroke pathway. The reduction in door-in to referral time indicates faster image interpretation and referral for EVT. There was an indication of an increased proportion of patients regaining independent function after EVT. However, this should be interpreted with caution given the small sample size. Larger, prospective studies and further systematic real-world evaluation are needed to demonstrate the widespread generalisability of these findings.

## 1 Introduction

Endovascular thrombectomy (EVT) is an established treatment for ischaemic stroke patients with large vessel occlusion (LVO) and has been shown in numerous studies to improve patient outcomes (1). Although selected patients may benefit from EVT up to 24 h after the onset of stroke, the benefit of treatment diminishes with increasing time between the onset of symptoms and treatment (2).

UK services to provide EVT are built around a ‘drip and ship’ model where those patients presenting at acute stroke centers (ASCs) within a geographical region, who are eligible for EVT, are transferred urgently to a comprehensive stroke center (CSC) after intravenous thrombolytic therapy, if indicated. The drip-and-ship model inevitably introduces a delay in EVT time to treatment for patients presenting to an ASC compared to those who present directly to a CSC.

**Figure 1:**
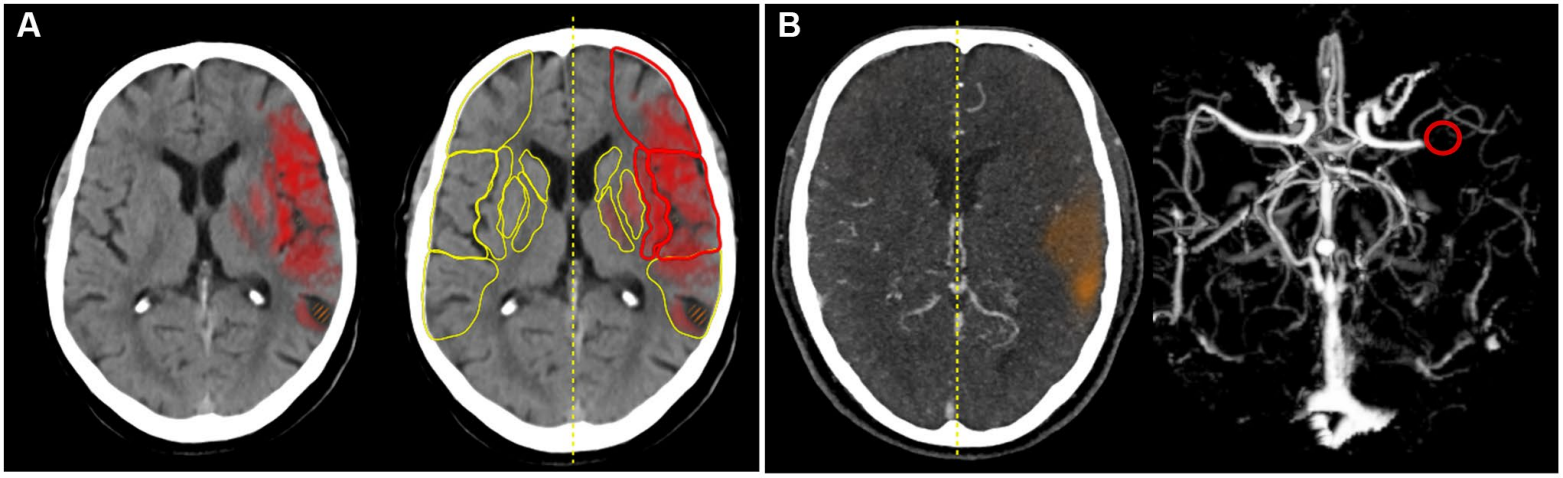
**(A)** e-ASPECTS. The heat map indicates early CT changes in NCCT and **(B)** e-CTA. The red circle indicates LVO in the CTA.

Early identification of LVO and timely treatment are critical to achieving optimal clinical outcomes, particularly when patients require transfer from an ASC to a CSC. In a drip-and-ship model, for transferred patients to achieve the most benefit, the door-in-door-out (DIDO) time at an ASC should be less than 60 min (3, 4). One factor that has been identified as preventing this is a lack of expertise to quickly diagnose an LVO at the ASC, especially at times when expert staff are less likely to be available. Timely and accurate interpretation of imaging has been identified as an element of the patient pathway where an artificial intelligence (AI) decision support tool may be used to improve decision-making and expedite care (5–7).

e-Stroke (Brainomix, United Kingdom) is a software medical device for processing CT scans for stroke patients. It uses AI to analyse stroke CT brain image data to provide automated detection of specific clinically validated imaging features (8, 9), including identification and quantification of early ischaemic change and hemorrhage, and identification of large vessel occlusion (10). The version used in this study comprised two separate image assessment modules of e-Stroke: e-ASPECTS, and e-CTA for non-contrast CT (NCCT) and CT angiography (CTA), respectively (Figure 1).

In this study, we aimed to evaluate the impact of the introduction of e-Stroke into the hyperacute stroke pathway at a representative ASC in the United Kingdom, assessing changes over time in relevant time metrics and clinical endpoints. We used STrengthening the Reporting of OBservational Studies in Epidemiology (STROBE) criteria to report its findings (11).

## 2 Materials and methods

A retrospective cohort study design was used to analyse the impact of e-Stroke before and after it was deployed into the stroke pathway at an ASC in the UK on 1 March 2020. All patients who were referred and underwent EVT were eligible and included in the analysis. The pre-e-Stroke cohort included patients referred for and underwent EVT from 01 January 2019 to 29 February 2020, and the post-e-Stroke cohort included patients from 01 March 2020 to 31 March 2021. We measured the following pre-specified endpoints: door-in-door-out time (DIDO), broken down into door-in-to-referral time (DTR), referral to acceptance time (RTA), acceptance-to-door-out time, and mRS at 3 months and dichotomised mRS 0–2 and mRS 5–6 between groups.

The data on age, sex, admission National Institute of Health Stroke Scale (NIHSS), and comorbidity were extracted from the Stroke Sentinel National Audit Program (SSNAP) and the local stroke registry. As this was a service evaluation at a single-center where routinely collected anonymised data were utilised, a separate ethical approval was not sought. All patients who were referred for EVT from the center were included in the registry, and the data on time-stamps and mRS at 3 months were collected prospectively. No major changes to the stroke pathway were made during the study period at this center other than the implementation of e-Stroke and remote decision-making facilitated by e-Stroke during the first wave of COVID-19.

### 2.1 Stroke pathway

The study ASC serves a population of approximately 600,000. Patients presenting with acute ischaemic stroke who are eligible for EVT are referred to one of the two regional CSCs, depending on the EVT service hours in the CSCs. Before the patient arrives at the emergency department (ED), the ambulance crew pre-alerts the ED team at the ASC for any suspected acute stroke patients, which triggers an acute stroke call to the local stroke team. The stroke team meets the patient on arrival at the ED, undertakes a clinical review, and then the patient is transferred to the CT unit for, NCCT and CTA.

The stroke team is led by a stroke specialist clinician during daytime hours or an emergency department physician after hours and at weekends, with stroke specialists available remotely for advice as required. All stroke specialists are consultant physicians with specialist training in stroke medicine or neurology. The team also includes a stroke specialist nurse at all hours. A trainee doctor and/or physician associate also support the team.

**Figure 2:**
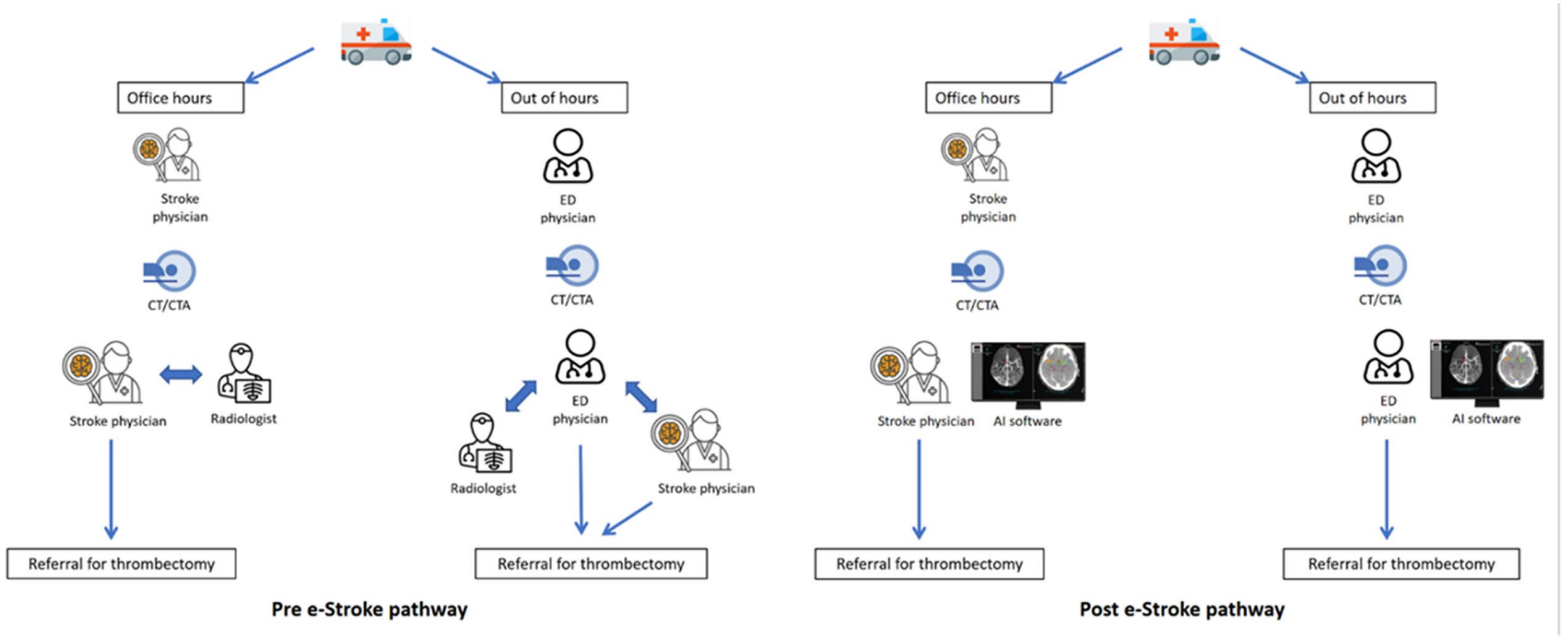
Pre- and post-e-Stroke pathway.

NCCT images are reviewed by a stroke specialist or an ED physician. However, not all stroke specialists or ED physicians have expertise in reviewing CTA images, and a radiologist review is often sought to confirm findings. Prior to e-Stroke implementation, the specialists had to use a laptop computer to access CT images remotely, and the images were transferred to CSCs via the picture archiving and communication system (PACS) or image exporting portal (IEP). Potentially EVT-eligible patients are referred to the on-call stroke specialist at the CSC and accepted after reviewing the images and eligibility. Subsequently, an ambulance transfer is arranged from the ASC to the CSC. In the meantime, eligible patients are given intravenous thrombolysis (Figure 2).

### 2.2 Introduction of e-Stroke

e-Stroke was implemented in March 2020. Acute stroke images (NCCT and CTA) were directly sent to the e-Stroke server from the CT scanner in parallel to being sent to PACS (Figure 2). e-Stroke result outputs were available on the PACS image viewer as well as on a mobile app, typically in less than 2 min from scan acquisition. In addition, one of the CSCs (CSC1) was linked via an integrated cloud-based image worklist to enable immediate review of e-Stroke images. The other CSC (CSC2) was able to view the e-Stroke images through image-sharing functionality via the e-Stroke app. The software was implemented as part of a proactive quality improvement initiative.

Mandatory face-to-face training was provided to all the clinical users before they were given access to the e-Stroke software. Additional training was provided to radiographers. Brainomix also supported a virtual training platform for user onboarding.

The pre-hospital alert system, the composition of the acute stroke team, and other elements of the pre-existing protocol for the stroke pathway were not changed during the study period, except for stroke specialists making treatment decisions remotely for stroke patients admitted to the ED with suspected symptoms of COVID-19 during the first wave of the pandemic. The remote communication was facilitated by instant WhatsApp imaging between the stroke nurse and stroke consultant group. The legal assurance by the Information Commissioners’ Office to the NHS England for adopting digital technologies for information sharing during COVID-19 supported this change (12).

### 2.3 Statistical analysis

Door-in-door-out, door-in to referral, referral to acceptance, and acceptance to door-out time comparisons before and after the introduction of AI-based decision support were performed using Welch’s t-test. To assess clinical outcomes, both shift and dichotomised analyses were used for the modified Rankin Scale (mRS) at 3 months, in keeping with standard practice. The distribution of mRS scores was compared using the Cochran–Mantel–Haenszel test. Proportional odds model regression was used for mRS shift odds ratios, confidence intervals, and adjustment for age and NIHSS. Dichotomised mRS 0–2, mRS 5–6, and baseline characteristics were compared between groups with Fisher’s exact test. All analyses were performed in R version 3.6.3 (13) and figures were drawn using ggplot2.

## 3 Results

During the 14-month period from 01 January 2019 to 29 February 2020, 846 patients were admitted to the ASC with acute stroke and 22 were referred to a CSC for EVT (pre-e-Stroke cohort). Of which, 19 patients received EVT. During the 12-month period following e-Stroke deployment (1 March 2020 to 31 March 2021), 785 patients were admitted with acute stroke and 25 were referred for EVT (post-e-Stroke cohort). Of which, 21 patients underwent EVT. The EVT referral rate in the pre- and post-e-Stroke cohorts was 2.6% vs. 3.2%, respectively (*p* = 0.55).

In the post-e-Stroke cohort, 13 of 25 (52%) patients were transferred to CSC1, which was linked via a cloud-based e-Stroke image sharing network. In the pre-e-Stroke cohort, 10 of 22 (45%) patients were transferred to CSC1. This difference was not statistically significant (*p* = 0.77).

Baseline patient characteristics are shown in Table 1. In the post-e-Stroke cohort, patients were more frequently older and had slightly less severe stroke severity. Although there were numerically more patients with vertebrobasilar occlusion in the pre-e-Stroke cohort, this difference is not statistically significant.

**Table 1:** Patient characteristics in pre- and post-e-Stroke cohorts.

	Pre-e-Stroke (*N* = 22)	Post-e-Stroke (*N* = 25)	*p*-value
Age (mean ± SD)	67 ± 16	72 ± 14	0.26
Sex (males)	8 (38%)	13 (52%)	0.38
NIHSS (median ± IQR)	20 ± 3	18 ± 9	0.14
Hypertension	10 (45%)	11 (44%)	1
Atrial fibrillation	9 (41%)	11 (44%)	1
Diabetes	1 (5%)	5 (20%)	0.19
Heart failure	2 (9%)	4 (16%)	0.67
Smoking	3 (14%)	1 (4%)	0.33
Previous stroke/TIA	3 (14%)	4 (16%)	1
ASPECTS (median and interquartile range)	8 (1)	9 (1)	0.79
pc-ASPECTS (median and interquartile range)	8 (4)	10 (0)	0.53
Occlusion site			0.56
ICA	1	4	
ICA/M1	1	2	
M1	12	15	
M2	3	3	
PCA	1	0	
Basilar artery	2	1	
Vertebral artery	2	0	

**Figure 3:**
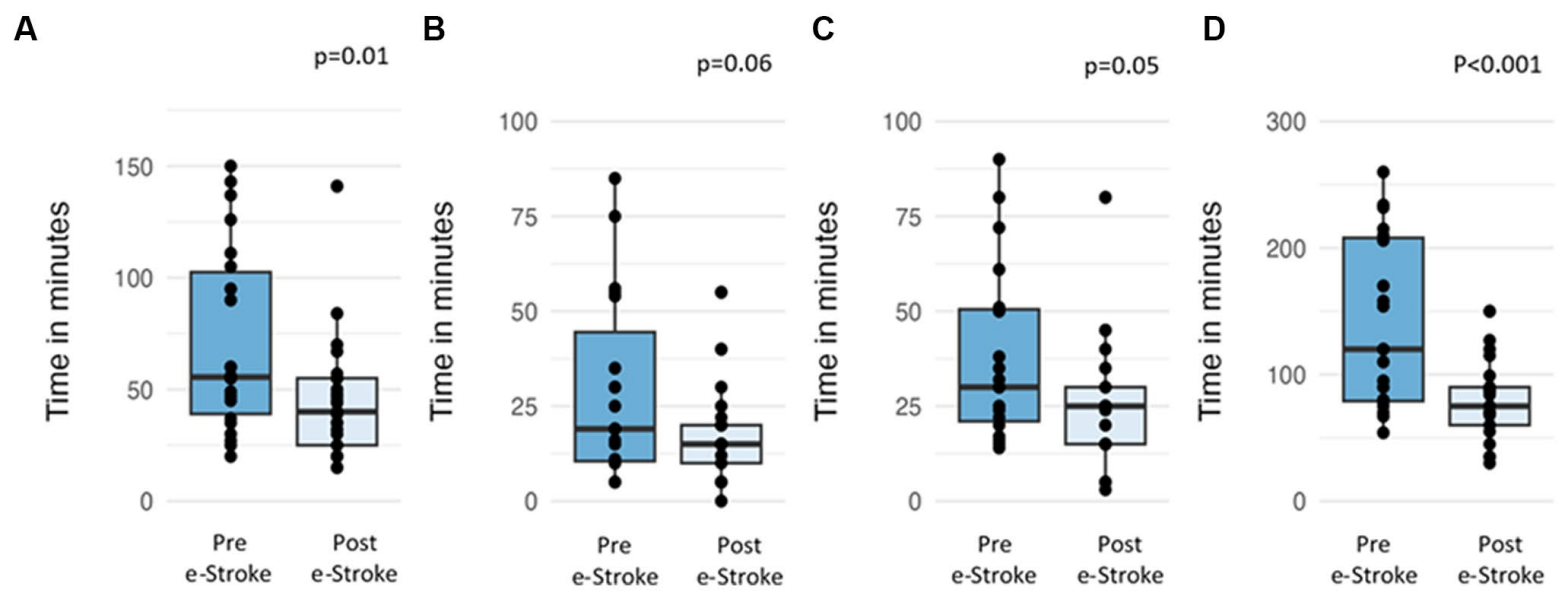
Time differences in the pre- and post-e-Stroke cohorts; **(A)** door-in to referral, **(B)** referral to acceptance, **(C)** acceptance to door out, and **(D)** door-in-door-out.

The mean treatment decision time differences between pre- and post-e-Stroke cohorts are as follows (Figure 3): door-in to referral time reduced from 71 min to 44 min (difference 27 min, 95% CI 47.4–5.4 min, *p* = 0.01), referral to acceptance time reduced from 29 min to 17 min (difference 12 min, 95% CI 24.9–0.6 min, *p* = 0.06), acceptance to door-out time reduced from 38 to 25 min (difference 13 min, 95% CI 26.4–0.19, *p* = 0.053), and door-in door-out (DIDO) time reduced from 141 min to 79 min (difference 62 min, 95% CI 96.9–26.8 min, *p* < 0.001).

**Figure 4:**
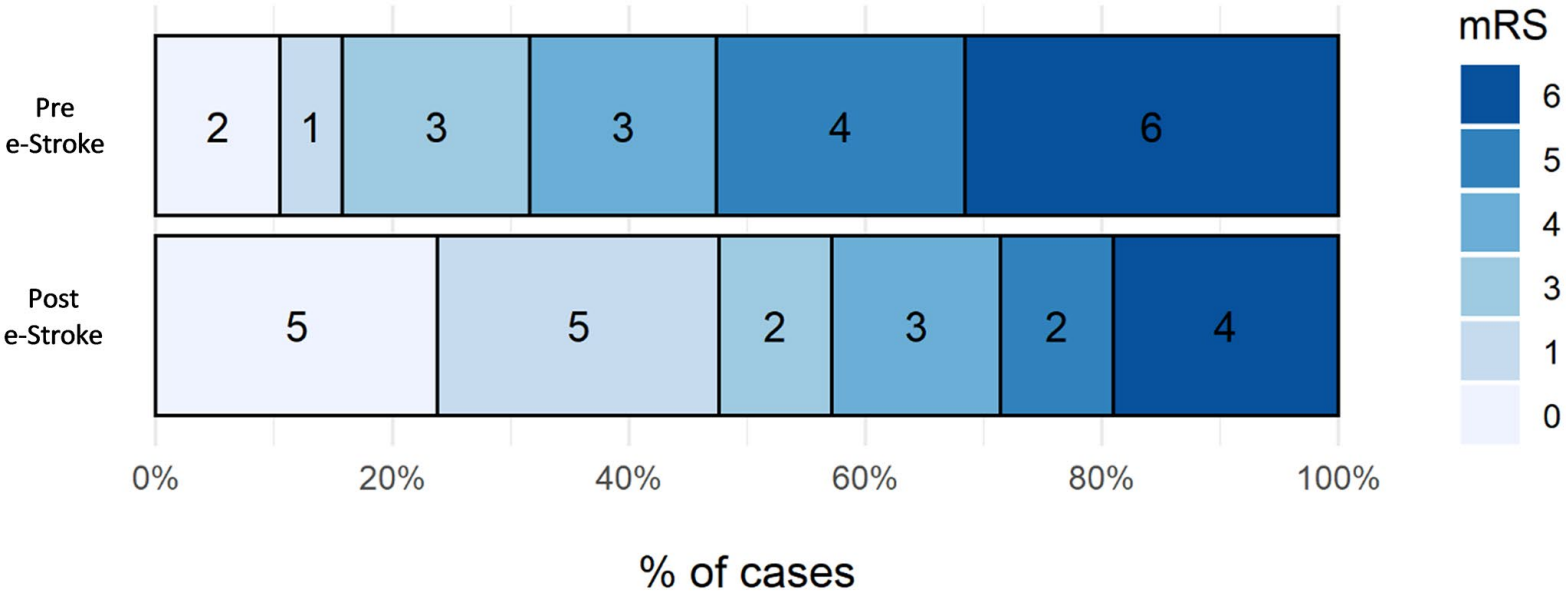
Three-month mRS shift analysis of the patients who received EVT pre- and post-e-Stroke implementation, 19 and 21 patients, respectively.

The mRS shift analysis of the patients who received EVT pre- and post-e-Stroke implementation, (19 and 21 patients, respectively) was not statistically significant, with only a trend towards improved outcomes following AI implementation. The adjusted (age and NIHSS) odds ratio for mRS ordinal shift analysis at 3 months was 3.14 (95% CI 0.99–10.51, *p* = 0.06) (Figure 4). Dichotomised mRS 0–2 at 3 months was achieved by 16% vs. 48% of patients for pre- and post-e-Stroke cohorts, respectively (*p* = 0.046); there were no cases with mRS 2. There was no significant difference in dichotomised mRS 5–6 at 3 months (53% before vs. 29% following implementation; *p* = 0.20) or in mortality (32% before vs. 19% following implementation, *p* = 0.47).

## 4 Discussion

Overall, in this exploratory study, we found an improvement in important time and patient flow metrics, particularly door-in-door-out time, after the implementation of the e-Stroke software. The ordinal shift analysis of mRS at 3 months did not reach significance. However, the dichotomised mRS analysis showed a trend towards improved clinical outcomes. The implementation of e-Stroke did not have a significant impact on EVT referral rates at the study ASC.

### 4.1 Time metrics

The results show a significant reduction in the mean DIDO time of 62 min, from 141 min to 79 min after the introduction of e-Stroke (95% CI 96.9–26.8 min, *p* < 0.001). As described above, no other significant changes occurred within the stroke pathway at the ASC during this time. Furthermore, the proportion of patients referred to each CSC was almost equal. Therefore, the reduction in DIDO time can be attributed to the improved efficiency of the existing patient assessment and referral pathway. The breakdown of the time segments that comprise the DIDO time shows that the impact was primarily driven by a reduction in door-to-referral time, likely reflecting improved patient identification at the ASC facilitated by e-Stroke.

The ambulance waiting time for secondary transfers can also affect the DIDO time. Our data show that the mean acceptance to door-out time reduced from 38 to 25 min (95% CI 26.4–0.19, *p* = 0.053). This trend may be explained by enabling faster secondary transfers when ambulance crews had not left the ASC by the time of the decision, providing availability for quicker secondary transfers. However, this information was not collected in the study.

The study period covers the pre- and post-COVID-19 pandemic. According to the Health Foundation Trust’s data for the United Kingdom, during the first wave of the COVID-19 pandemic, ambulance waiting time, patients waiting for more than 4 hours for an ambulance and staff sickness were on the rise (14). Further, McConachie et al. (15) have reported a 27% reduction in the number of EVTs performed in the UK and process delays due to the need for patient testing and isolating during the first wave of COVID-19. Therefore, the COVID-19 pandemic is likely to have had a negative impact on the processes around acute stroke care. Nevertheless, our study demonstrates the improved efficacy of the pathway with the introduction of e-Stroke.

The study ASC is one of the high-performing stroke centers in the United Kingdom for delivering hyperacute stroke care. The center consistently achieved an A rating (the rating ranges from A – the highest to E – the lowest) in the national audit data (SSNAP), with an IVT rate of more than 20% (national average 11%) and a median door-to-needle time of 30 min (national average 55 min) (16). The significant reduction in DIDO time in our study shows that even in high-performing centers, significant improvements can be attained in the EVT referral pathway with the help of e-Stroke.

### 4.2 Clinical outcomes

Dichotomised mRS 0–2 at 3 months, showing the proportion of patients achieving functional independence, increased 3-fold in the post-e-Stroke cohort (16% vs. 48%, *p* = 0.046). There was also a trend for improvement in the mRS shift analysis, but this did not reach statistical significance. It is important to consider that in a complex multi-step stroke pathway, it is difficult to directly attribute improved outcomes to a single intervention. However, in a recent study, Flores et al. (17) have shown that in transferred patients undergoing EVT, DIDO has a significant impact on clinical outcomes. Therefore, the significant reduction in DIDO time in our study could be attributed to the trend observed in the mRS analysis.

This study is a real-world evaluation of the introduction of AI decision-aid software into a stroke pathway and its impact on clinically meaningful endpoints. Another study that evaluated the impact of e-Stroke in a stroke pathway demonstrated an increase in the rate and timing of thrombolysis but did not describe the impact on the broader patient pathway metrics (7). Our study reports patient outcomes linked to the implementation of AI software into a National Health Service (NHS) stroke pathway as well as the process metrics.

In the transformation of any complex clinical pathway, human factors play a key role in the adoption of the change. However, the perception, usage, or impact of e-Stroke at an individual clinician level was outside the scope of this study. Furthermore, an assumption was made that the e-Stroke was used similarly in every case, but it is unlikely to have happened in real life. All stroke scans were automatically analysed by e-Stroke software. Although it was an individual clinician’s decision whether the outputs were used, the cloud-linked images of AI output had been used during referral to CSC. Therefore, one could assume the e-Stroke would have been used in most cases.

Our study is limited by a relatively small sample size owing to the volume of transferred cases at our center, and will require validation in larger datasets. This study focused on the pathway in an ASC and did not consider procedural success, pathway efficiency at CSCs, and access to rehabilitation that contributes to patient outcomes. Despite the limitations of a single-center, retrospective design, we believe this study adds important evidence to help understand the potential benefits of AI software in an acute stroke pathway.

## 5 Conclusion

In this single-center study in the United Kingdom, the DIDO time significantly decreased since the introduction of e-Stroke decision support software into an ASC hyperacute stroke pathway. The reduction in door-in to referral time indicates faster image interpretation and referral for EVT. There was an indication of an increased proportion of patients regaining independent function after EVT. However, this should be interpreted with caution, given the small sample size. Larger, prospective studies and further systematic real-world evaluations are needed to demonstrate the widespread generalisability of these findings.

## Data availability statement

The original contributions presented in the study are included in the article/supplementary material, further inquiries can be directed to the corresponding author.

## Ethics statement

Ethical review and approval was not required for the study on human participants in accordance with the local legislation and institutional requirements. Written informed consent from the patients/participants or patients/participants' legal guardian/next of kin was not required to participate in this study in accordance with the national legislation and the institutional requirements.

## Author contributions

KN: Conceptualization, Data curation, Formal analysis, Investigation, Methodology, Validation, Writing – original draft, Writing – review & editing. AN: Data curation, Formal analysis, Methodology, Validation, Writing – review & editing. JB: Writing – review & editing. GF: Writing – review & editing. ZW: Writing – review & editing. DM: Data curation, Writing – review & editing. GH: Writing – review & editing.
